# Book review: a clinician’s guide to binge eating disorder - edited by June Alexander, Andrea B. Goldschmidt & Daniel Le Grange

**DOI:** 10.1186/2050-2974-1-24

**Published:** 2013-08-30

**Authors:** Brooke Adam

**Affiliations:** 1 Boden Institute of Obesity, Nutrition, Exercise and Eating Disorders, University of Sydney, Camperdown NSW 2006, Australia

## 

The book aims to educate clinicians about the triggers and behaviours characteristic of binge eating disorder (BED); and translate that knowledge into treatment approaches across medical and psychological frameworks. It is written by a wide range of skilled clinicians, researchers and advocates, for clinicians of any discipline, at any level. The book covers binge eating across the community, from child and adolescents through to adults; male and female.

The book is divided into two complementary themes; the first section of the book begins with a search for causes, presenting the reader with a collection of chapters outlining the etiology of binge eating. Section one not only provides an overview of current research, but clearly explains the clinical implications of research findings. The second section presents solutions across a vast range; from surgical intervention and pharmacotherapy treatments, through to psychological interventions and prevention. Case studies are presented throughout many chapters of the book, which I found particularly useful to highlight the real-life implications of BED; helpful to the novice or expert clinician.

The foreword presents a brief overview of the recent decision to include BED into the Diagnostic and Statistical Manual, Fifth Edition (DSM-5). From the outset, this text highlights the complexities of BED, both in terms of the relationship between known precipitants of binge eating, as well as the gaps in current knowledge; for example, whether the two defining features of a binge episode, the sense of a lack of control and consuming an objectively large amount of food, are equally important in the classification of BED and bulimia nervosa.

Section one eloquently outlines recent research on the genetic and environmental factors contributing to the onset of BED. It covers an impressive range of topics and consistently couches research findings within a clinical context, beginning with an examination of the overlap between BED and obesity and the crucial importance of screening for ethnicity and socio-economic background when assessing for and treating BED. The somewhat controversial, emerging research on the neurological systems and genetic factors in the development of BED is outlined in a clear and accessible style in chapter three. Bulik and Trace skillfully highlight how this information can be used in a clinical setting to educate and empower individuals in treatment for BED.

Sections also consider the etiology of binge eating from a developmental perspective; current understanding of childhood trauma and the risk factor it presents for the onset of eating disorders and other psychiatric comorbidities, presenting valuable practical suggestions for clinicians working with persons with BED who report childhood trauma.

An epidemiological approach is taken in considering the common comorbidities seen in BED in chapter six. This is a refreshing discussion with the aim of facilitating the integration of research into practice. Mond, Star and Hay explore the extensive comorbidities between eating disorders and other mental health diagnoses with clear implications for clinicians and public health. It is one of the few chapters to address gender considerations and differences in treatment and service utilisation, and quality of life impairment beyond diagnosed psychiatric comorbidities.

Chapters also examine the well-established risk factor and maintaining factor of body image disturbance in eating disorders, and related cognitive-behavioural treatment approaches; an eloquent, well-considered discussion of the importance of science in the therapy room; and why evidence-based treatment is critical from the perspective of an individual in recovery from an eating disorder.

The reader arrives at section two, an overview of treatment, with a solid theoretical and empirical foundation in mind. Section two opens with an introductory chapter by Judith Banker, which sets the scene for the rest of the book. One of the core challenges in treating BED is reflected upon; that is, until the inclusion of BED in the DSM-5, individuals with BED were lumped into the category of eating disorder not otherwise specified (EDNOS) - the category commonly excluded from research trials, yet the most widely-used eating disorder diagnosis. Research is building on effective treatments for BED, however clinicians often adapt manualised treatments that have been tested in research trials. An informed argument is presented for an iterative process to bridge the existing gap between research, clinical experience and application to clinical practice.

Chapters in section two consistently and effectively highlight the bridge between research and clinical practice, by presenting a range of treatments for BED. A succinct overview of the skills and tools needed for appropriate screening, assessment and diagnosis of BED is presented. For clinicians interested in Dialectical Behaviour Therapy (DBT), chapter ten outlines modified DBT for BED. This chapter will be of key interest for any clinician working with individuals with BED who don’t respond to more ‘traditional’ treatments e.g. cognitive behavior therapy (CBT), or for clinicians who work with multi-impulsive individuals presenting for treatment.

A discussion of the frameworks of CBT and interpersonal psychotherapy (IPT) applied to BED are reviewed, alongside a review of controlled treatment trials of pharmacotherapy. Limitations of pharmacologic treatments are discussed in an accessible style. Both topics present salient information for any clinician working in this area. Subsequent chapters cover binge eating in children and adolescents; bariatric surgery and the large gaps in current understanding of the relationship between pre-surgery binge eating and post-surgery outcomes; and an examination of behavioural weight loss (BWL) for BED, with an insightful discussion of the importance of considering socio-cultural factors as well as individuals’ expectations of weight loss before commencing treatment.

Section two also considers a novel treatment approach in the form of intuitive eating and movement (IEM), which works on integrating all aspects of the ‘self’ as a way to build a more compassionate relationship with one’s body. A thought-provoking chapter highlights the necessity for prevention-based interventions with adolescents, aimed at targeting both BED and obesity, through targeting pathways such as early-onset dieting; followed by a section outlining the personal costs of BED, the importance of reducing stigma, and future directions for prevention, treatment and recovery.

Finally the book finishes with a chapter that highlights the responsibility of clinicians to harness effective, evidence-based treatments that support, educate and treat individuals with BED. It repeats a core theme presented in many chapters throughout the book: to effectively treat BED we must continue to understand, conceptualise and treat it as a discreet mental health disorder, and bring it into public understanding in the same way anorexia nervosa and bulimia nervosa have been, rather than grouping individuals with BED with those seeking treatment for obesity and weight loss. In the afterword, the importance of advocacy and collaboration at a community, clinical and research level is discussed. I found the afterword to be particularly thought-provoking and it highlighted just how important the recognition of the BED diagnosis has been at many levels in the community.

In summary, this text provides clinicians with a broad, evidence-based framework that offers a map for various treatment approaches, depending on the background of the reader, as well as a sound overview of current research, including gaps in the literature that require further exploration. In reading this book I was impressed with how extensive the existing body of research is, and how quickly it is advancing, given the relatively short period of time since the inclusion of BED as a provisional diagnosis in the DSM-IV-TR.

I found this book to be thought-provoking, insightful and well-written. The impressive research, clinical and personal experiences of the authors provide the reader with complementary perspectives on this potentially debilitating eating disorder. The case studies presented through most chapters highlight core clinical issues in a manner that is difficult to do achieve in pure research texts or papers. It has been written with the clinician firmly in mind, who will benefit from using this text irrespective of whether they are a novice therapist eager to learn more about BED, or a highly experienced clinician keeping up-to-date with current understanding and treatment approaches.

## Author short bio

Brooke Adam is a Clinical Psychologist and PhD Candidate at the Royal Prince Alfred Hospital and the Boden Institute of Obesity, Nutrition, Exercise and Eating Disorders, and Honorary Associate at the University of Sydney. She is the eating disorder Service Coordinator for Sydney/South West Sydney Local Health Districts. Brooke specialises in the inpatient and outpatient treatment of eating disorders, with a special research interest in Binge Eating Disorder.

## Competing interests

The author declares that she has no competing interests.

